# Across the edge: Spatial segregation drives community structure in tri‐trophic multilayer networks at a forest–grassland edge

**DOI:** 10.1111/1365-2656.70120

**Published:** 2025-08-28

**Authors:** Henrique Negrello‐Oliveira, José Tovar‐Marquez, Milton de Souza Mendonça Júnior

**Affiliations:** ^1^ Graduate Program in Ecology Federal University of Rio Grande do Sul Porto Alegre Rio Grande do Sul Brazil

**Keywords:** antagonistic networks, edge effects, habitat connectivity, host–parasitoid, modularity, network dissimilarity, predator–prey, trophic levels

## Abstract

1. Examining spillover between habitat boundaries offers a key opportunity to understand how neighbouring habitats may affect each other. Although extensively studied, ecological responses at forest–grassland edges are variable across trophic levels and their underlying interactions. Thus, tackling the subject from a multitrophic perspective may yield valuable insights into how energy may flow across forest–grassland edges.
2. We asked whether a forest–grassland edge functions as an ecological barrier or a continuum for species interactions across space and trophic levels. We also examined whether species influence in the network is better explained by their distribution across the edge (spatial structure) or by their connections with other species (modular structure).
3. We studied a tri‐trophic (prey–consumer–parasitoid) antagonistic system at Atlantic Forest and Pampa Grassland edges, arranged in fire‐prone mosaics in southern Brazil. Using network dissimilarity and multilayer approaches, we investigated species and trophic‐level contributions to connectivity across the spatial/modular landscape by sampling cavity‐nesting hymenopterans and their interactions across a distance gradient from the habitat edge
4. We found spatially segregated modules confined to each habitat, indicating that the edge likely functions as an ecological barrier. Network dissimilarity peaked in cross‐habitat comparisons, reinforcing the separation between forest and grassland ecosystems. While all trophic levels were less adaptable to shifts between habitats and modules, they showed greater adaptability across spatial strata within each habitat. The main factor determining species influence throughout the network was their ability to move across spatial layers, although trophic‐level and habitat subgroups also responded to other variables. Cross‐edge species had greater influence in connecting habitats internally than in serving as energy pathways between them.
5. Our findings reveal that Atlantic Forest‐Pampa Grassland edges likely constitute an ecological barrier network‐wise. However, edge effects increasing interaction richness and abundance may highlight the importance of edge proximity to key species promoting within‐habitat network cohesion. Our results highlight how network dynamics may span across habitat edges with significant species turnover, calling for active conservation strategies to prevent forest encroachment and maintain grassland habitats—while recognising that disturbances within the roughly 40‐m edge effects zone could potentially cascade inward, influencing species and interactions beyond the edge.

## Graphical abstract

This study reveals how forest–grassland edges act as ecological barriers that shape tri‐trophic interaction networks. Using multilayer network theory, it uncovers how species influence and biodiversity patterns emerge across spatial layers, offering novel insights into edge‐driven community assembly and conservation in fire‐prone mosaics.

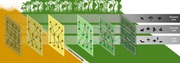

## Resumo

1. Estudar o transbordamento entre limites de habitats é crucial para entender como ambientes vizinhos podem influenciar‐se mutuamente. Embora já bastante investigadas, as respostas ecológicas nas bordas entre floresta e campo variam entre níveis tróficos e suas interações. Assim, abordar o tema sob uma perspectiva multitrófica pode oferecer perspectivas valiosas sobre como a energia flui entre floresta e campo.
2. Buscamos compreender se a borda entre ambientes florestais e campestres atua como uma barreira ou como um contínuo ecológico, dadas as interações entre espécies ao longo do espaço e entre diferentes níveis tróficos. Também investigamos se a influência das espécies na rede é melhor explicada por sua distribuição ao longo da borda (estrutura espacial) ou por suas conexões com outras espécies (estrutura modular).
3. Para isso, estudamos um sistema antagonístico tritrófico (presa–consumidor–parasitoide) em áreas de ecótono Pampa‐Mata Atlântica, arranjadas em mosaicos suscetíveis ao fogo no sul do Brasil. Utilizando abordagens de dissimilaridade e redes multicamadas, avaliamos a contribuição de espécies e níveis tróficos para a conectividade espacial e modular, por meio da amostragem de himenópteros que nidificam em cavidades e suas interações ao longo de um gradiente de distância em relação à borda.
4. Identificamos módulos segregados espacialmente e restritos a cada habitat, sugerindo que a borda funciona como uma barreira ecológica. A dissimilaridade das redes foi maior nas comparações entre habitats, reforçando a separação entre floresta e campo. Todos os níveis tróficos mostraram baixa capacidade de transição entre habitats e módulos, mas apresentaram maior flexibilidade dentro de cada habitat. A influência das espécies na rede foi determinada principalmente por sua capacidade de persistir em diferentes camadas espaciais, embora grupos definidos por nível trófico e habitat também tenham respondido a outros fatores. As espécies que ocorrem em ambos os habitats contribuíram mais para conectar cada habitat internamente do que para atuar como vias de fluxo energético entre eles.
5. Nossos resultados indicam que áreas de ecótono Pampa‐Mata Atlântica tendem a funcionar como barreiras ecológicas. No entanto, os efeitos de borda que aumentam a riqueza e a abundância de interações destacam a importância dessa faixa para espécies‐chave que mantêm a coesão das redes dentro dos habitats. Esses achados mostram que a dinâmica das redes ecológicas pode se estender para além das bordas, acompanhada por forte substituição de espécies, o que reforça a necessidade de estratégias de conservação que evitem a expansão florestal sobre os campos. Além disso, é importante reconhecer que perturbações dentro da zona de efeito de borda, estimada em cerca de 40 metros, podem se propagar para o interior e afetar espécies e interações para além dessa faixa.

## 1 INTRODUCTION

At the boundaries where contrasting ecosystems meet, habitat edges highlight the balance between stability and change within ecological systems (Porensky & Young, 2013; Ries et al., 2004). Edge effects, conveyed as the changes in species distributions in response to habitat boundaries, rank among the most studied topics in the ecological literature, given the ubiquity of ecological edges in an increasingly fragmented world (Fahrig, 2017). Such pervasiveness triggers a plethora of responses from species' communities and their interactions, where edges can harbour amalgamations of species from both adjacent habitats, rendering compositionally unique assemblages (Bond & Parr, 2010; Erdős et al., 2013), as well as sustaining edge‐specialist species that contribute to the functional uniqueness of boundary zones within heterogeneous landscapes (Fletcher et al., 2018). For arthropods at forest–grassland edges, for example, both responses are seen (Magura et al., 2001; Martin & Major, 2001; Wimp & Murphy, 2021), likely due to various arrangements of intrinsic factors like mobility (Caitano et al., 2020) and extrinsic factors such as prey/host availability and landscape context (Thies et al., 2003; Toma & Mendonça Jr, 2013). Therefore, understanding the spatial scale at which each of these factors operates may yield valuable insights into how energy may flow across habitat edges.

Focusing on networks of species interactions may offer a more reliable measure of change across habitat edges, as interactions can shift even when species composition remains unchanged (Tylianakis et al., 2010). Multilayer networks are particularly useful for addressing spatially discriminated interactive communities, allowing for network dynamics to span over multiple spatial strata and thus creating a more realistic view of ecological processes (Hutchinson et al., 2019; Olesen et al., 2011; Pilosof et al., 2017). While species turnover may increase dissimilarity across edges (Erdős et al., 2013; Tylianakis & Morris, 2017), nonspatial network topologies such as modules can also contribute to network change across habitats (Hervías‐Parejo et al., 2023). Modules are defined as subsets of species interacting preferably among themselves due to ecological/evolutionary processes that may lead to local specialisation and niche partitioning (Cordeiro et al., 2020; Guimerà et al., 2010; Olesen et al., 2007). Given that modularity is hypothesised to enhance network stability by containing the propagation of disturbances within modular structures (Landi et al., 2018; Thébault & Fontaine, 2010), examining the spatial distribution of modules at habitat edges provides valuable insights into how they may function as ecological boundaries or continua (Peralta et al., 2017).

Individual species may also play a key role in edge connectivity, linking networks in different habitats and thus promoting cross‐habitat cohesion (Frost et al., 2016; McCann et al., 2005). This role can be addressed by quantifying a species' (i.e. ‘node’) potential influence within a network, through centrality indices such as closeness, which measures a node's ability to influence others and vice versa (Freeman, 1978). Previous studies have demonstrated the usefulness of centrality metrics to identify key species across landscape mosaics, allowing for the detection of spatially explicit drivers of community change (Avon & Bergès, 2016; Estrada & Bodin, 2008). Furthermore, their disproportionately high influence can be evidenced by a faster breakdown of network structure when actively removed, potentially even leading to community fragmentation (Albert et al., 2000; Memmott et al., 2004). However, in networks characterised by structural complexity, such as modularity in antagonistic systems, species influence may also arise from its importance within local modules and/or their ability to connect them (Blüthgen et al., 2008). Therefore, investigating whether species' influence is primarily shaped by their role within local modules/habitats or by bridging dynamics across them is essential to reveal the underlying mechanisms driving landscape permeability across metacommunities (Hagen et al., 2012).

Structurally heterogeneous habitats can provide distinct pathways for energy to flow, as environmental conditions may determine where disturbances cascade to within a trophic chain (Pocock et al., 2012). In forest–grassland edge habitats, productivity and structural complexity seem to be good predictors of arthropod multitrophic responses (Loyola & Martins, 2008). While lower trophic‐level species respond more strongly to vegetation structure and plant diversity (Scherber et al., 2010), higher trophic‐level species are more likely to shift between spatially segregated modules (Caitano et al., 2020; Timóteo et al., 2018), which may lead to spillover effects between adjacent habitats (Frost et al., 2015; Rand et al., 2006). Furthermore, although body size may increase in higher trophic levels, which is correlated with dispersal capacity (Cohen et al., 1993; Gathmann & Tscharntke, 2002), so does trophic generality, potentially relieving those species of habitat‐specific foraging limitations (Vejřík et al., 2023). This pattern of contrasting spatial responses across trophic levels may underscore the compartmentalisation of species' effects at habitat edges, highlighting the need to explore the relative significance of top‐down and bottom‐up effects in multitrophic interactions.

In this study, we set out to examine the extent to which a forest–grassland edge functions as an ecological barrier or continuum by sampling antagonistic tritrophic interactions at increasing distances from the edge inside each habitat. Specifically, we aimed to: (1) determine how species interaction networks change in response to the habitat edge; (2) identify closely interacting groups of species among trophic levels and spatial layers; (3) assess each trophic level's influence over the network and versatility to habitat and module shifts; and (4) analyse the extent to which species' network influence is driven by their influence within local modules/habitats and/or their capacity to connect those structures. If the habitat edge works as an ecological continuum, we expect that (i) cross‐habitat network comparisons shall be more similar at the edge, with increasing dissimilarity as other equidistant layers are compared (e.g. intermediate forest vs. intermediate grassland, interior forest vs. interior grassland, etc.), (ii) module density (i.e. number of modules) will be higher at habitat edges due to edge effects of shared species/modules between habitats and (iii) cross‐edge (CE) species influence will come primarily from their ability to connect different habitats. If it otherwise constitutes a barrier, we expect cross‐habitat comparisons to be highly dissimilar yet uniform across equidistant layers in each habitat, module density to be consistent across within‐habitat spatial strata, and CE species influence to derive from their independent effect within each habitat. Finally, to determine the structural drivers of species influence across habitats, we ask: What is a better predictor of a species' influence across the habitat axis—the extent to which it is embedded within network modules or its ability to persist across spatial layers?

## 2 MATERIALS AND METHODS

### 2.1 Study region and system

The study was conducted on three hills (equally separated by 12 km) in rural areas of Porto Alegre City (southern Brazil) within or near protected areas: *Morro Santana* (170 m above sea level, 30°4′0.45″ S, 51°7′44.97″ W), *Morro do Osso* (150 m, 30°6′58.76″ S, 51°14′6.67″ W) and *Morro São Pedro* (200 m, 30°10′26.34″ S, 51°6′53.90″ W). All sites contain forest–grassland hard edge mosaics (Subtropical Atlantic Forest and Pampa Grassland remnants), mediated by fire regimes since at least the early Holocene (Behling & Pillar, 2007).

We sampled above‐ground cavity‐nesting hymenopterans, their prey and parasitoids as our tritrophic system. By nesting in artificial cavities such as bamboo stems or hollow twigs, both their offspring and paralysed prey remain ‘trapped’ and may be attacked by parasitoid species, revealing up to three trophic levels and their underlying interactions (consumer–prey and host–parasitoid; Krombein, 1967). These species show high diversity and abundance across both forests and grasslands (Buschini & Woiski, 2008), yet their movement patterns are often highly localised. Individuals usually forage within a few metres of their nests, indicating strong site fidelity—and foraging trip duration, used as a proxy for distance, also tends to be short for both bees and spider‐hunting wasps (Hennessy et al., 2021; Klein, Steffan‐Dewenter, & Tscharntke, 2004). Moreover, small‐scale habitat structure and proximity to resources strongly influence foraging behaviour (Klein, Tylianakis, et al., 2004; Steffan‐Dewenter, 2002; Tscharntke et al., 1998), and their prey species are also known to respond strongly to habitat edges (Rodrigues et al., 2014). This renders cavity‐nesting hymenopterans particularly suitable for exploring how both vertical (trophic‐level) and horizontal (habitat‐level) network dynamics cascade across forest–grassland edges.

### 2.2 Sampling protocol

**FIGURE 1:**
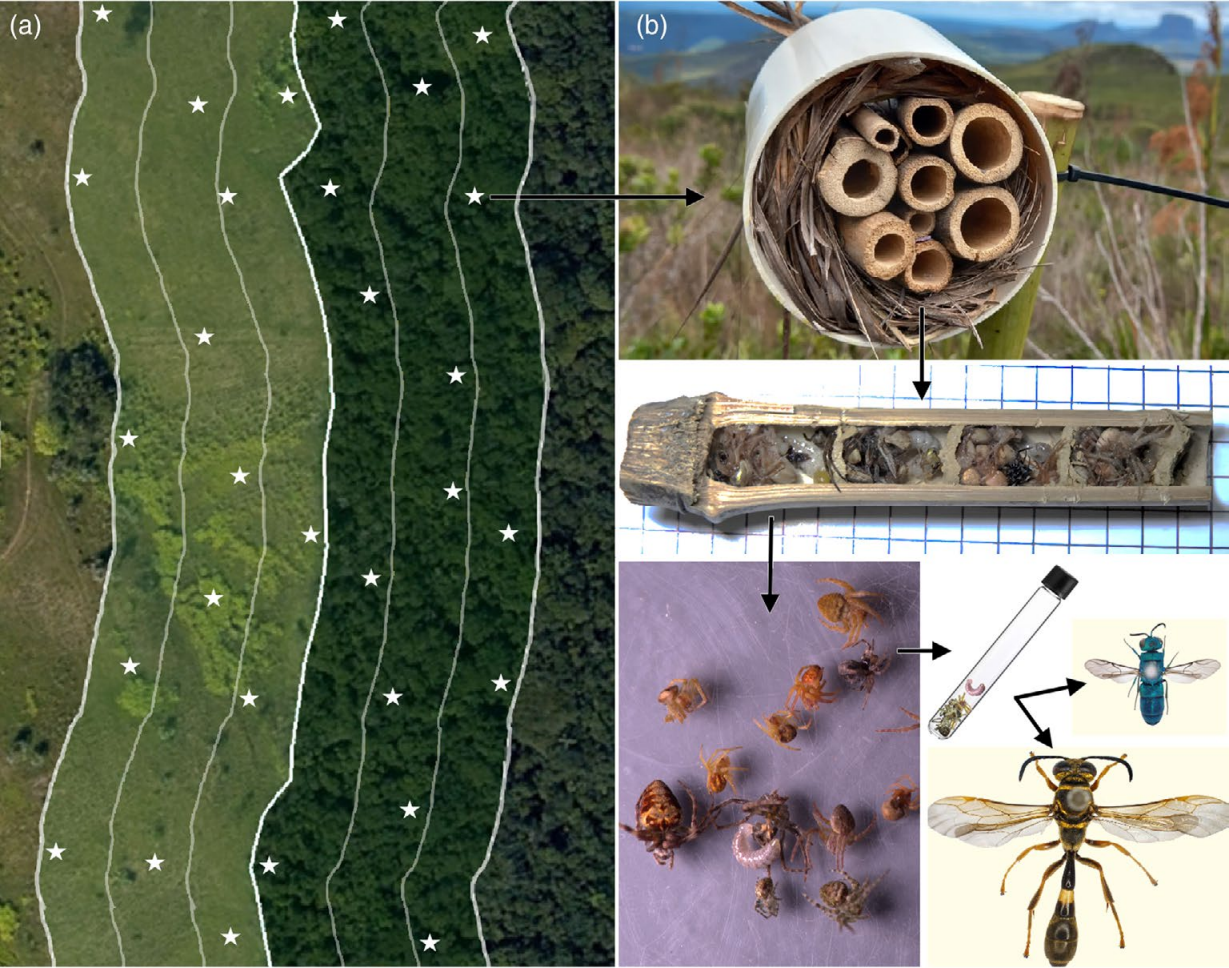
Schematic representation of the sampling design (a) and trap‐nesting method (b). Bamboo stems mimicking natural nesting cavities are occupied and opened to reveal cells containing paralysed prey. Rearing larvae to maturity yields cavity‐nesting or parasitoid species, thus revealing consumer–prey and host–parasitoid interactions. Photographs: Henrique Negrello‐Oliveira.

Within each hill, we established the same six sampling treatments (spatial layers), relative to the distance from the edge between the habitats: forest and grassland edge (F1/G1, 0–20 m inside each habitat), forest/grassland intermediate (F2/G2, 20–40 m) and forest/grassland interior (F3/G3, 40–60 m). We adopted 20 m strata as a biologically meaningful resolution based on foraging constraints and site fidelity in cavity‐nesting hymenopterans, allowing us to detect fine‐scale variation in colonisation and species interactions. In each layer of each site, we randomly installed five 1.5 m tall bamboo poles (at least 15 m apart) containing 15 trap‐nests each (Figure 1a). Trap‐nests consisted of 10 cm long bamboo stalks (openings ranging evenly from 5 to 15 mm), used by the species as a nesting resource. In total, 90 poles were installed across all three sites, totalling 1350 traps.

Trap‐nests were inspected fortnightly from October 2022 to March 2023. Occupied traps were replaced by identical empty ones and taken to the laboratory. There, traps were opened longitudinally, where we (1) recorded the number of cells (individual chambers) per nest, (2) if present, carefully removed all prey content to be photographed (consumer–prey interactions) and (3) placed them in glass vials (kept at ambient outdoor temperature to mimic field conditions) until adult cavity‐nesting species or their parasitoids emerged (host–parasitoid interactions; Figure 1b). We randomly selected 20% of the cells as reference material, with their contents stored in alcohol for further taxonomic determination. Once identified, those IDs were extended to identical prey items in photographs. Reared wasps and parasitoids were frozen, pinned and identified with expert assistance. All material was deposited in the collections of UFRGS (cavity‐nesting wasps, dipteran parasitoids, bees, spiders and caterpillars), UFPEL (crickets) and UFES (hymenopteran parasitoids).

All methods were conducted in accordance with institutional guidelines and national regulations. Data collection within protected areas was performed under research permits issued by the management teams of Parque Natural Morro do Osso (PNMO‐001/2022) and Refúgio de Vida Silvestre São Pedro (ReViSSP‐003/2022).

### 2.3 Spatial multilayer networks

**FIGURE 2:**
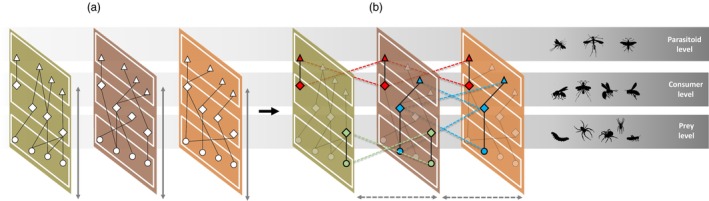
Schematic diagram of the multilayer architecture. Intralayer edges (a) connect species (nodes) between adjacent trophic levels (white boxes) within the same spatial layer (coloured boxes). Interlayer edges (b) also connect species across trophic levels, but between different spatial layers—provided that the same interaction pair was observed in both layers.

Merging data from all sites, we set up a multilayer network regarding the interactions between species across habitats. This network was composed of (1) a set of spatial layers, where interactions took place (the sampling treatments), (2) a set of state nodes (regarding the manifestation of a species in a given layer), (3) a set of weighted edges, representing connections between state nodes, either within the same spatial layer (intralayer edges) or across different spatial layers (interlayer edges) and (4) a set of trophic levels, positioning each species in the food web (as prey, consumer or parasitoid; Figure 2). Trophic level‐wise, we treated all species attacking trap nests as parasitoid‐level species (parasitoids, kleptoparasites, etc.) and all species occupying nests as consumer‐level species (predatory wasps and bees), for the sake of simplicity. A detailed explanation of the edge structure is provided in Supporting Information S1.

### 2.4 Network spatial dissimilarity

To evaluate network‐level change across space, we calculated interaction dissimilarity between spatial layers using the ‘betalink’ *R* package (Poisot et al., 2012; R Core Team, 2024), both within (edge‐intermediate, intermediate‐interior and edge‐interior) and between habitats (edge‐edge, intermediate‐intermediate and interior‐interior). The main indices were species composition dissimilarity *β*_S_ (difference in species identities between layers) and network dissimilarity *β*_WN_ (difference in interaction overlap). The latter was partitioned into dissimilarity in network structure due to interaction turnover *β*_OS_ (rewiring, i.e. interactions occurring in only one layer despite both partners being present) and species turnover *β*_ST_ (i.e. interactions not shared due to species not co‐occurring). All indices (*β*_S_, *β*_WN_, *β*_OS_ and *β*_ST_) range from 0 (complete similarity) to 1 (complete dissimilarity).

### 2.5 Species module affiliation and trophic level versatility

We applied the Infomap search algorithm (Edler et al., 2017) to detect modular flows in our multilayer structure, thus revealing highly interactive groups of species across trophic levels and spatial layers. The flow is modelled by dropping a random walker at any given node and recording its movement, which reflects the direction and weight of the edges themselves. The map equation then converts the flow rates within and between modules to an information‐theoretic modular description measure of the random walker's movement on the network (*L* value, measured in bits). We used the function ‘*run_infomap_multilayer*’ (1000 trials), available at the ‘infomapecology’ R package (Farage et al., 2021), using the ‘rawdir’ flow model (quantitative edge weights) to obtain a list with module affiliations for each state node. To detect how both bipartite networks (consumer–prey and host–parasitoid) were contributing to the observed modularity, we shuffled intralayer links within each layer while preserving connectance and marginal totals, followed by a new calculation of interlayer links over the shuffled layers. For the consumer–prey network, only intralayer edges of consumer‐level species belonging to the same dietary guild were randomised to avoid type 1 errors from ‘forbidden interactions’ among known dietary specialists (i.e. cricket‐hunter wasps would not hunt spiders, spider‐hunters would not prey on caterpillars, etc.). We repeated this process 1000 times (each iteration with 1000 algorithm trials), resulting in 10^6^ total runs for each trophic level pair. To address species versatility to module changes, we calculated adjustability metrics, which are the proportion of species from a given trophic level that switch modules at least once across layers (Pilosof et al., 2017). We extended this concept to calculate versatility among layers (species presence in more than one layer) and between habitats (presence in both habitats).

### 2.6 Species influence within/across habitats and trophic levels

To assess each species' influence over the network, we calculated species closeness centrality, which describes the mean minimal number of steps (edges) needed to reach all other nodes in the network—ranging from 0 (no capacity to affect other species) to 1 (full capacity, all nodes reachable within one step). This was done both within each habitat (forest/grassland) and over the entire multilayer structure (i.e. full network), in order to capture species' potential to affect others in each of those structures (Supporting Information S2). We compared within‐habitat centrality values between forest and grassland (including all species) to assess which habitat was more interconnected. To make values comparable, we rescaled them relative to the full network, ensuring that differences in network size across habitats did not bias the centrality estimates (see Supporting Information S3). We also compared centrality values across trophic levels and between single‐habitat (SH) and CE species—both within and across habitats—to examine whether these subgroupings differed in their influence across each network structure (i.e. forest, grassland, and the full network). All comparisons were performed using *t*‐tests and ANOVAs, followed by post hoc Tukey tests. Finally, by partitioning the centrality values of each CE species—weighing their values within each habitat against their values over the full network—we were also able to assess those species' potential to ‘bridge’ dynamics between habitats, reflecting their relative influence in each (Supporting Information S3).

### 2.7 Drivers of species influence

To address species' influence across the spatial/modular landscape, we computed weight (*z*‐scores) and connectivity (participation coefficients) metrics proposed by Guimerà and Nunes Amaral (2005) and later refined by Hackett et al. (2019) to allow for landscape‐scale measures. Specifically, we looked at two metrics for each species' modular dynamics: within‐module weight, which reflects a species' overall dominance within affiliated modules (i.e. the proportion of all interactions in a module involving the species), and cross‐module connectivity, which captures how evenly its interactions are distributed across those modules. Similarly, we computed two metrics for each species' spatial dynamics: within‐layer weight, which reflects its overall dominance within the spatial layers it occupied (i.e. the proportion of all interactions in a layer involving the species), and cross‐layer connectivity, which captures how evenly its interactions are distributed across those layers (Supporting Information S4).

We fitted hierarchical generalised linear mixed models to assess whether the spatial or modular structure of the network—represented by weight and connectivity metrics as predictor variables—better explained the variation in species influence, with full‐network closeness centrality as the response variable. We tested four groups of predictors to explain species influence in the network: a spatial hypothesis, accounting for spatial dynamics as the main predictors (within‐ and cross‐layer metrics); a modular hypothesis, for modular dynamics (within‐ and cross‐module); a global hypothesis (containing all predictors); and a null hypothesis (no predictors). We considered species habitat (SH grassland, SH forest and CE species) and trophic level (prey/consumer/parasitoid) as subgroups, given a posteriori differences in closeness found across those groupings. We used Akaike Information Criterion (AICc) to select the model with the best fit to our data, and applied a 95% confidence interval to account for predictor variability (Supporting Information S5).

## 3 RESULTS

Across the habitat axis, we collected 574 occupied trap‐nests containing 1924 individual cells (3.16 ± 1.89 cells per nest), revealing 290 unique interactions among 142 species (15 parasitoid, 9 consumer and 118 prey‐level species) and 2248 total interactions (2028 consumer–prey, 220 host–parasitoid). Consumer‐level species included spider‐hunters (*Trypoxylon* spp., *Auplopus subaurarius*), caterpillar‐hunters (*Monobia angulosa* and *Zethus adonis*), cricket‐hunters (*Isodontia costipennis*) and bees (*Megachile* sp.). Spider‐hunters were key contributors to the network, accounting for the majority of both consumer–prey (1799 interactions; 88% of the total) and host–parasitoid interactions (193 interactions; 87% total).

Total interaction abundance increased near habitat edges, peaking in edge layers (*grassland*: G1—462, G2—378, G3—233; *forest*: F1—515, F2—483 and F3—177), though interaction richness was highest at intermediate layers (*grassland*: G1—45, G2—48 and G3—21; *forest*: F1—62, F2—75 and F3—39). The forest habitat was overall more diverse, with more species (91 vs. 71), higher interaction richness (176 vs. 114 pairs) and greater abundance (1175 vs. 1073 links). Grasslands had slightly more consumer–prey interactions (1052 vs. 976), while forests had more host–parasitoid links (199 vs. 21). The habitats were compositionally distinct, with only 20 species shared between habitats (14%).

### 3.1 Network spatial dissimilarity

**FIGURE 3:**
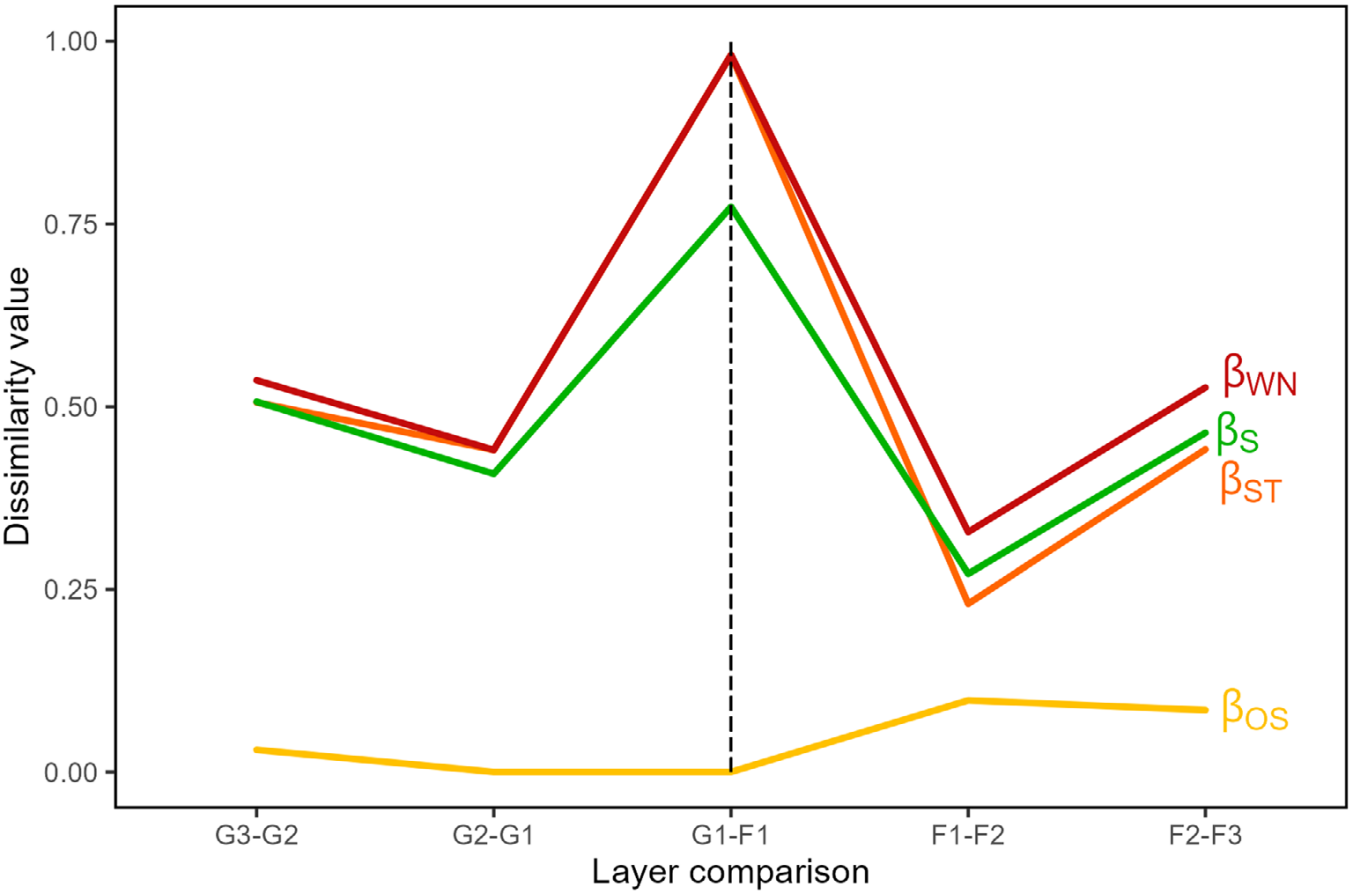
Species composition dissimilarity (*β*_S_, green), network dissimilarity (*β*_WN_, dark red), network dissimilarity due to species turnover (*β*_ST_, orange) and to rewiring (*β*_OS_, yellow) across adjacent‐layer comparisons. Layer pairs are shown on the *x*‐axis, with the dashed line indicating the habitat edge.

Species composition dissimilarity (*β*_S_) was stable (~50%) in intermediate‐interior and edge‐interior comparisons but decreased in edge‐intermediate comparisons (forest: 27%, grassland: 40%) (Figure 3). Network dissimilarity (*β*_WN_) and species turnover (*β*_ST_) followed a similar trend, emphasising species turnover as the primary driver of network spatial change. Rewiring (*β*_OS_) was minimal in grasslands and edge comparisons but increased deeper in the forest. Dissimilarity peaked in CE comparisons (*β*_S_: ~75%, *β*_WN_: ~100%), further stressing network segregation between habitats. Edge‐edge comparisons were virtually identical to other equidistant pairs (intermediate‐intermediate and interior‐interior), revealing that edge layers are not more alike than spatial pairs farther apart (Supporting Information S6).

### 3.2 Trophic and spatial arrangement of multilayer modules

**FIGURE 4:**
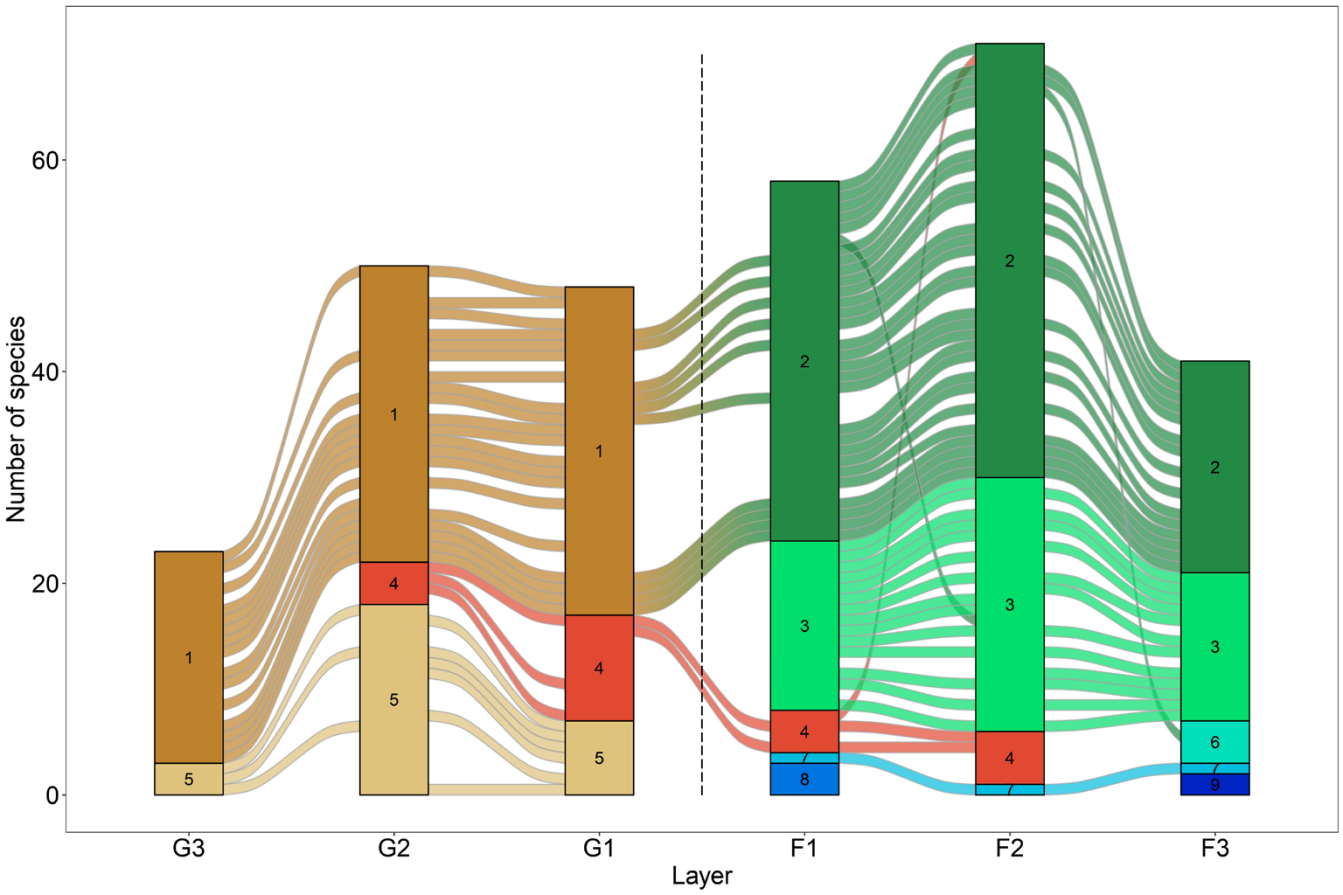
Alluvial diagram of multilayer module distribution across the habitat axis. Modules (rectangles) cluster species, with height showing module size. The dashed line sets the habitat edge. Colours represent module types: brown (grassland‐only), red (cross‐edge) and blue/green (forest‐only). Although species can flow from further layers, alluvials (species movement between modules in different layers) are only exhibited between neighbouring layers, to adapt the 3D structure to a 2D representation.

**FIGURE 5:**
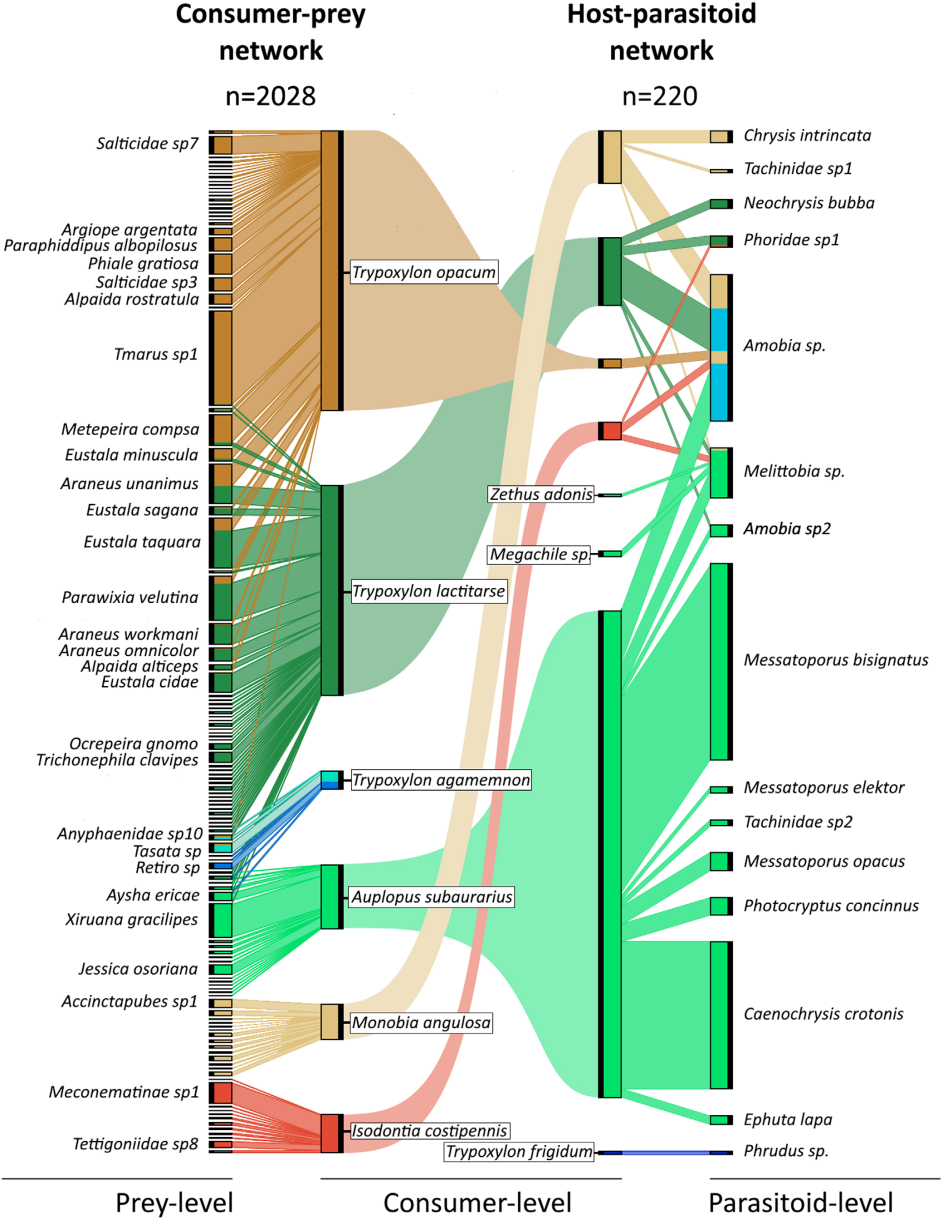
Metaweb (spatially aggregated network) of consumer–prey (left) and host–parasitoid (right) interactions. Boxes represent species abundances, and straight links indicate the interaction frequency between each pair, reflecting overall network size. Sinuous links connecting left and right networks are merely graphical, to connect consumer‐level species' appearances as consumers of prey (left) or hosts to parasitoids (right). Only prey species with *n* > 15 are labelled. Colours indicate module affiliation, as in Figure 4.

We identified nine multilayer modules across the habitat axis (Figures 4 and 5; Supporting Information S7; map equation *L* value = 2.83). Most modules were centred around consumer species and confined within the spatial range of each habitat (*grassland*—2 modules, *forest*—6 modules); however, one module (#4, in red in Figure 4) was able to span across the intermediate and edge layers of both habitats. While several spider‐hunter modules prevailed in the forest, the grassland habitat was functionally more diverse, with caterpillar or cricket‐hunter modules appearing in all layers (although with a single module from each). There was a single module (#7) containing three state nodes of one parasitoid (*Amobia* sp.), due to this species being too evenly linked to several consumer‐level species. Spider‐hunter wasp *Trypoxylon agamemnon* pertained to different modules (#6 and #8) at the forest edge/interior, due to its interaction turnover between the two layers. Only two compartments (disconnected components) were found over the entire aggregated tri‐trophic network, one with 2 (*Phrudus* sp. parasitoid and *Trypoxylon frigidum* spider‐hunter, module #9) and the other with the remaining 140 species. We found that consumer–prey interactions contributed significantly to our observed modularity (randomised *L* value = 3.13 ± 0.04, *p* = 0.001), while host–parasitoid interactions did not (randomised *L* value = 2.82 ± 0.01, *p* = 0.852).

All trophic levels exhibited low values of habitat versatility, highlighting the spatial segregation effect between habitats (Supporting Information S8). The prey level showed the highest value (14%), likely due to 14 of cricket species being captured in both habitats. Moreover, the cricket‐hunter *I. costipennis* was highly influential to this index within its consumer level, as the only species able to cross the habitat edge. Layer versatility peaked at 77% in the consumer level, closely followed by parasitoids (73%), showing the overall high tolerance for different spatial strata within these trophic levels, as long as shifts were occurring within the same habitat. Although relatively low, the parasitoid level showed the highest value of module versatility (20%), underscoring the adaptability of some parasitoids in interacting with different hosts.

### 3.3 Trophic and habitat‐level network influence

**FIGURE 6:**
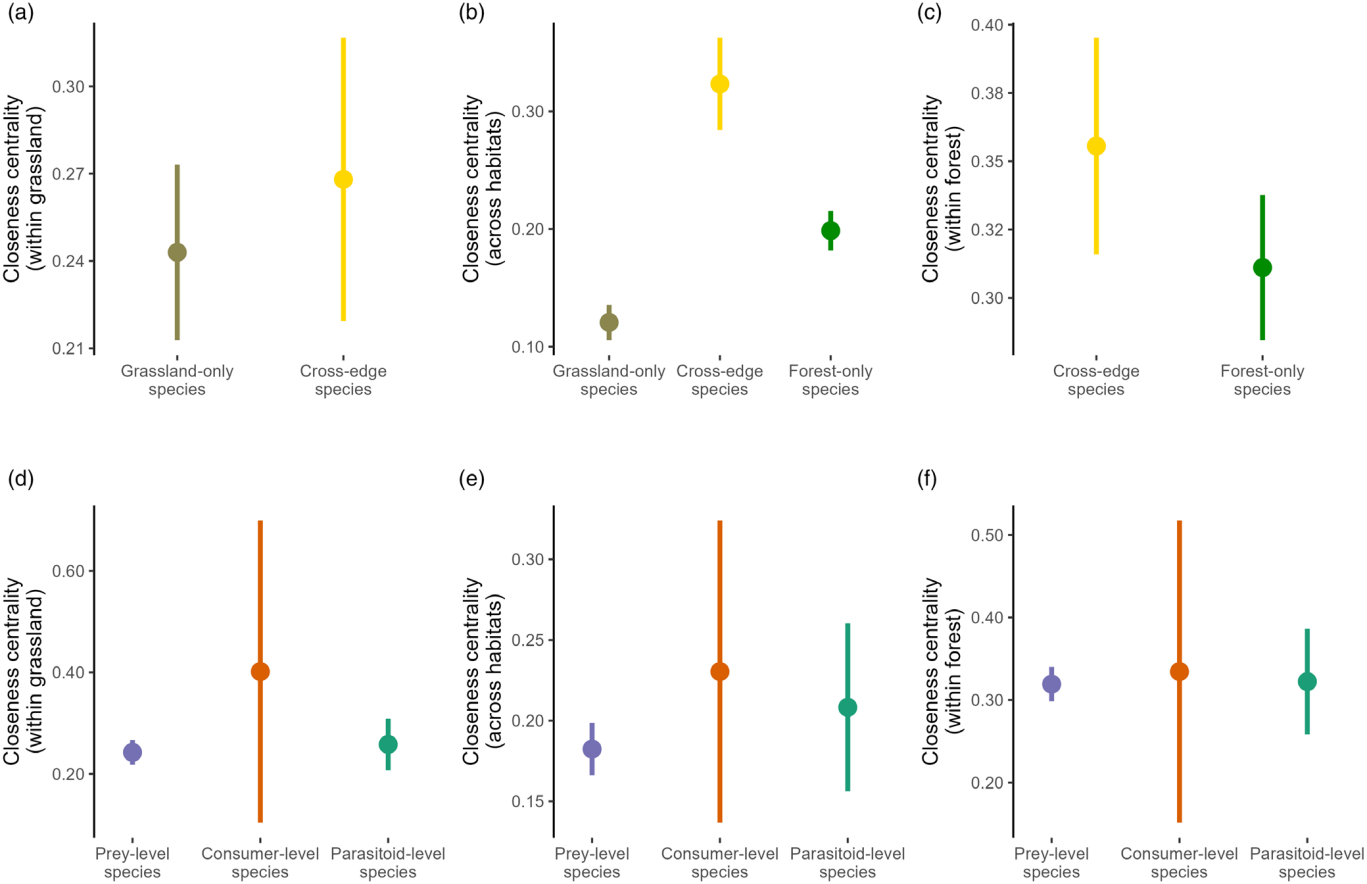
Differences in closeness centrality between single‐habitat and cross‐edge species (a–c) and among trophic levels (d–f). Comparisons are shown within the grassland habitat (a, d), across habitats (b, e) and within forest (c, f). Points represent group means, and error bars indicate 95% confidence intervals.

**FIGURE 7:**
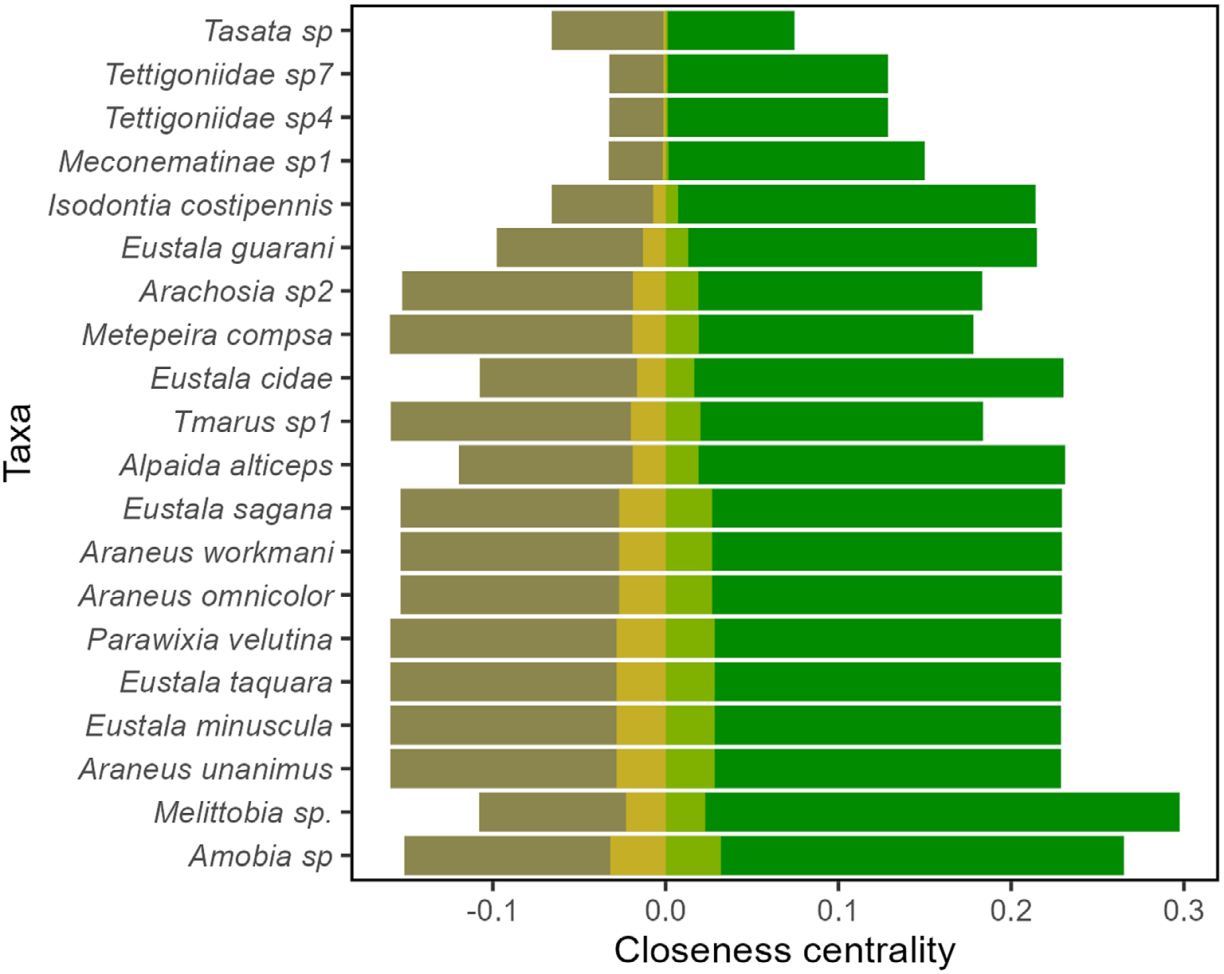
Cross‐edge species closeness centralities over the entire network. Values are partitioned regarding species influence within forest (green, positive values), grassland (light brown, negative values) and bridging potential across habitats (golden).

Different species ranked highest in centrality in each habitat, highlighting habitat spatial segregation (Supporting Information S9). Forest species had higher closeness centrality (0.32 ± 0.11) than grassland species (0.25 ± 0.10), revealing a more interconnected network structure in the forest (*t* = 4.06, df = 150.8, *p* < 0.001). CE species were not significantly more influential than SH species within each habitat (Forest: *t* = 1.829, df = 37.79, *p* = 0.075; Grassland: *t* = 0.858, df = 34.43, *p* = 0.396; Figure 6a–c). However, CE species (0.32 ± 0.08) were more influential than both SH species (Forest: 0.19 ± 0.07, Grassland: 0.12 ± 0.05) across the full network (*F*_(2,139)_ = 62.8, *η* ^2^ = 0.47, *p* < 0.001). The effect of CE species as habitat connectors was minimal (<0.1; Figure 7), revealing that their higher influence stemmed primarily from their independent effect within each habitat.

We found that species closeness differed among trophic levels within the grassland habitat (*F*_(2,68)_ = 3.2, *η* ^2^ = 0.08, *p*‐value = 0.046), but not within the forest (*F*_(2,88)_ = 0.06, *η* ^2^ < 0.01, *p* = 0.942) or across the full network (*F*_(2,139)_ = 1.4, *η* ^2^ = 0.02, *p* = 0.239; Figure 6c–f). Within grassland, post hoc comparisons revealed that consumer‐level species (0.40 ± 0.26) had a significantly higher closeness than prey species (0.24 ± 0.09), which in turn was similar to parasitoids (0.25 ± 0.05). This suggests that while top‐down mechanisms are likely to be shaping consumer–prey interactions, top‐down/bottom‐up effects may have a more equal impact in host–parasitoid links within this habitat. Similarly, the lack of significant comparisons across the full network (prey: 0.18 ± 0.08, consumer: 0.23 ± 0.14, parasitoid: 0.20 ± 0.10) and within the forest habitat (prey: 0.31 ± 0.08, consumer: 0.33 ± 0.24, parasitoid: 0.32 ± 0.11) further evidences the balanced influence of top‐down and bottom‐up mechanisms driving consumer–prey/host–parasitoid interactions.

### 3.4 Drivers of species influence

**FIGURE 8:**
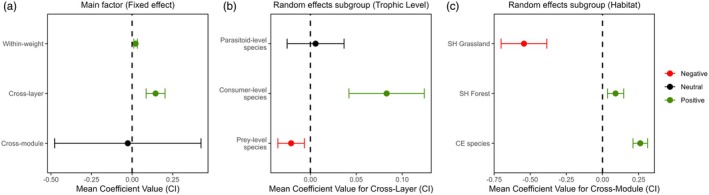
Slope coefficients and their respective confidence intervals (95%) regarding the relationship between species closeness centrality (influence) and spatial/modular connectivity metrics. Values were averaged across equally plausible models (ΔAICc <2) to extract the overall relationships between species influence and fixed effects (a). Random effects included group variation across habitats (b) and trophic levels (c). Effect significance was based on parameter consistency, and classified as neutral when confidence intervals included zero (black), positive when above zero (green) and negative when below (red).

The best fit was reached by a global model (all variables as fixed effects) with random intercepts and random slopes for cross‐module connectivity among habitats and cross‐layer connectivity among trophic levels, accounting for 83.7% of variance in species influence (Supporting Information S10). Overall, we found that a more uniform distribution of interactions across spatial layers (cross‐layer connectivity) was the main factor driving species influence across the network (Figure 8a). However, while consumer‐level species with greater spatial connectivity presented higher influence, for prey species, it had a negative effect (Figure 8b). Parasitoids, on the other hand, showed no significant response to cross‐layer connectivity, suggesting that their influence is less dependent on how their interactions are distributed across spatial layers. Albeit positive, species importance within modules/spatial layers (within‐weight) had a smaller effect on species influences across the network.

While cross‐module connectivity (evenly distributed interactions across modules) did not exert a consistent overall effect on species influences, its impact varied across species groupings: positive for CE and SH forest species but negative for SH grassland species (Figure 8c). This further highlights the more disjointed nature of grassland modules, contrasting with the forest habitat's more interconnected structure. CE species cross‐habitat influence, while limited, may represent a critical link between habitat‐segregated modules, explaining their greater influence relative to their SH counterparts.

## 4 DISCUSSION

Confirming our ‘barrier’ hypothesis, we found that CE network comparisons did not increase in dissimilarity as comparisons of equivalent layers got farther into each habitat. The lack of such a trend reveals how the hard habitat edge likely acts as an ecological barrier, preventing most species and interactions from enduring the sudden biotic/abiotic shift to a neighbouring habitat, even at close edge distances. Conversely, the relatively lower dissimilarity found within each habitat stresses the homogeneity of those spatial strata, where species appear largely unaffected by the presence of the adjacent habitat. However, the sudden drop in dissimilarity found at both habitats' edge‐intermediate comparisons may also indicate edge effects to some extent, prompting a ~40 m ‘edge effects zone’ that, despite being highly similar to other spatial strata, could be subjected to different biotic/abiotic regimes regarding species interactions (Cadenasso & Pickett, 2001; Macfadyen & Muller, 2013).

Although abiotic pressures such as humidity and insolation vary greatly from grassland to forested habitats and are known to affect arthropod communities directly (Prather et al., 2020), biotic factors such as habitat primary productivity may also determine functional responses in multi‐trophic interactions (Albert et al., 2022). Previous studies have reported that Pampas grasslands exhibit lower net primary productivity compared to forest habitats—particularly those with high biomass and productivity, such as the subtropical Atlantic Forest (Oliveira et al., 2022; Vassallo et al., 2013; Wysiecki, 1993). This contrast might explain why the grassland habitat not only harboured fewer modules but also showed a higher dominance of primary consumers as prey (cricket and caterpillar modules) when compared to the forest, dominated by several modules containing higher trophic‐level prey species (spiders). Highly productive habitats better sustaining higher trophic‐chain species have been a well‐established phenomenon in the ecological literature (Abrams, 1993; Yee et al., 2007), a pattern also consistent with our finding of a higher frequency of host–parasitoid links in the forest habitat.

Module persistence over space further stresses our findings on interaction dissimilarity, revealing a rather inconspicuous spatial structure within each habitat but pronounced between them. Most modules span across several spatial layers within the same habitat, showing that species interactions within a habitat are weakly affected by the habitat edge, thus confirming our barrier hypothesis regarding module density at the habitat edge. A notable exception was *I. costipennis*, the only consumer‐level species able to cross the edge and establish nests in both habitats. Those results, along with the significant modularity found at the consumer–prey network, suggest the presence of some underlying mechanism shaping consumer–prey interactions. Niche partitioning towards foraging behaviour has been an extensively studied structuring driver of predatory wasp assemblages (Falcón‐Brindis et al., 2019; Moura et al., 2019) and may explain the spatial distribution of interactions among predatory consumer‐level species. Forest consumer‐level species, although mostly spider‐hunting wasps, shared very few prey links among themselves and even less so at the grassland, where a single species of each dietary guild was found (no shared consumer–prey links). Most consumer–prey shared interactions were registered among spatially segregated predators (e.g. *Trypoxylon lactitarse* and *Trypoxylon opacum*), which could also explain the intrusion of *I. costipennis*, an allegedly open‐canopy species (Buschini & Woiski, 2006), into the forest habitat, devoid of other cricket‐hunting species. However, it should be noted that our trap‐nesting method limits us to cavity‐nesting species, meaning the full extent of niche partitioning—particularly when considering ground‐nesting species—remains beyond the scope of this study.

The balanced network influence across trophic levels suggests an interplay between top‐down and bottom‐up mechanisms in maintaining network cohesion (Vidal & Murphy, 2018). However, the higher influence of consumer‐level species in grassland highlights the habitat's more compartmentalised network structure, with fewer prey/parasitoid species linking consumer‐centred modules and thus reducing the overall influence of grassland species. This is further emphasised by the impact of cross‐module connectivity on species influences: while forest species showed marginally positive relationships (probably due to the habitat's higher network cohesion), grassland species exhibited a strongly negative effect, suggesting that within‐module dynamics were likely the primary driver of species centralities in the grassland. The influence patterns of SH and CE species reinforce our ‘barrier’ hypothesis, demonstrating that even species occurring in both habitats do not effectively bridge energy flow between them. This suggests that the compartmentalised structure of networks across edges may constrain the expected role of shared species as conduits of energy flow between adjacent habitats, limiting their influence beyond their within‐habitat contributions (Lundberg & Moberg, 2003; Polis et al., 1997).

Despite not being significantly modular network‐wise, host–parasitoid interactions were highly specialised, with most parasitoids attacking a single consumer‐level species. Such restrictions in host ranges have long been suggested as an evolutionary path to overcome host defences (Futuyma & Moreno, 1988; Jaenike, 1978), though it may ‘lock’ parasitoids to their host spatial availability (Thies et al., 2003). By contrast, generalist parasitoids can be more mobile over spatial strata and host species (Fornoff et al., 2021; Rand & Tscharntke, 2007), thus increasing module and habitat versatility at this trophic level. Two key generalists in our system were the parasitoids *Amobia* sp. and *Melittobia* sp., which ranked among the most influential species and were the only parasitoid‐level taxa capable of crossing the habitat edge. This functional flexibility has been reported in previous studies (Lee & Kim, 2019; Spofford et al., 1989), underscoring their potential role as key ‘energy spreaders’ across modules, whether spatially segregated or not. In fact, the affiliation of the caterpillar‐hunting *Z. adonis* and bee *Megachile* sp. to the same module of spider‐hunter *A*. *subaurarius* (through shared interactions with *Melittobia*) may even suggest some level of apparent competition among seemingly isolated consumer‐level species, when considering their dietary guilds (Frost et al., 2015; Morris et al., 2004, 2005).

Our findings provide consistent evidence that although our forest–grassland edge constitutes an ecological barrier network‐wise, edge habitats may play a crucial role for species within each habitat, as indicated by edge effects increasing interaction abundance and richness near the edge. Moreover, a few species were pivotal to maintaining network cohesion within both habitats, a role that is especially critical to be understood in the Neotropics, where insect species that are still poorly known can be consequential to network structure. In fact, our work led not only to the first description of males for two parasitoid species previously known only from females (*Messatoporus elektor* and *Messatoporus opacus*; Aguiar et al., 2024), but also to the discovery of a new species, *Messatoporus bisignatus*—remarkably, the most abundant parasitoid in our samples. These results highlight the ecological relevance of forest–grassland edge habitats in southern Brazil, and active conservation strategies—such as prescribed burning or cattle grazing—may be necessary to preserve the Atlantic Forest–Grassland Pampa mosaics (Behling et al., 2007). If conservation management excludes disturbances such as the above, the current grassland patches are likely to disappear due to forest encroachment under the prevailing humid climate (Müller et al., 2012). While these regimes may be essential for grassland maintenance, it is also important to consider that disturbances within the ‘edge effects zone’—extending roughly 40 m from the habitat edge into each habitat—could potentially cascade into the interior, altering species interactions and community structure beyond the edge zone. Altogether, we underscore the importance of adaptive management strategies that not only apply disturbance‐based interventions with strategic foresight but also reconcile ecological preservation with the dynamic nature of these mosaic landscapes.

## AUTHOR CONTRIBUTIONS

Henrique Negrello‐Oliveira and Milton de Souza Mendonça Júnior conceived the ideas and sampling design; all authors collected the data; Henrique Negrello‐Oliveira and José Tovar‐Marquez identified specimens; Henrique Negrello‐Oliveira led the analyses and writing of the first draft; all authors contributed to writing and reviewing of following drafts, and gave final approval for publication.

## CONFLICT OF INTEREST STATEMENT

The authors declare no conflict of interest.

## STATEMENT ON INCLUSION

Our study is based in Latin America and benefits from the contributions of Latin American researchers from multiple countries, ensuring that local expertise, knowledge and perspectives are embedded in the research. Our work was developed within conservation areas, and the discovery of novel species contributes to a better understanding of regional biodiversity and supports efforts to secure financial resources for the protection of yet poorly known taxa. Whenever relevant, we have cited literature published by scientists from Latin America, ensuring that our study builds on their work and contributes to ongoing scientific efforts in the region.

## Supporting information


**Supporting Information S1.** Network edge definitions and weighting scheme for intra and interlayer connections.
**Supporting Information S2.** Computation of multilayer closeness centrality.
**Supporting Information S3.** Partitioning habitat contributions to closeness centrality and quantification of bridging potential.
**Supporting Information S4.** Within‐weight and cross‐connectivity metrics across spatial layers and interaction modules.
**Supporting Information S5.** Hypothesis families, model building, and selection procedure.
**Supporting Information S6.** Summary of species composition dissimilarity (*β*_S_), network dissimilarity (*β*_WN_) and network dissimilarity due to either species turnover (*β*_ST_) or rewiring (*β*_OS_) among and within habitat layer comparisons.
**Supporting Information S7.** Species distribution across the habitat axis, partitioned by trophic levels/species groups. Colours indicate module affiliation, as in Figure 4.
**Supporting Information S8.** Summary of versatility values (i.e. proportion of species switching modules, layers or habitats at least once) discriminated by species groups (italic values) within each trophic level (bold values).
**Supporting Information S9.** Closeness centralities of species in forest (*x* axis) and grassland (*y* axis).
**Supporting Information S10.** Summary of AICc classification for models with ΔAICc <2 (equally plausible).

## Data Availability

Data are available from the Dryad Digital Repository https://doi.org/10.5061/dryad.nzs7h4540 (Negrello‐Oliveira et al., 2025).
