# Supplementary material for: Across the edge: Spatial segregation drives community structure in tri‐trophic multilayer networks at a forest–grassland edge
Source: J Anim Ecol. 2025 Aug 28;94(11):2205–19. doi: 10.1111/1365-2656.70120 (PMC12586797; doi:10.1111/1365-2656.70120)
Supplement: Supplementary file 1 — Supporting Information S1. Network edge definitions and weighting scheme for intra and interlayer connections. Supporting Information S2. Computation of multilayer closeness centrality. Supporting Information S3. Partitioning habitat contributions to closeness centrality and quantification of bridging potential. Supporting Information S4. Within‐weight and cross‐connectivity metrics across spatial layers and interaction modules. Supporting Information S5. Hypothesis families, model building, and selection procedure. Supporting Information S6. Summary of species composition dissimilarity (β S), network dissimilarity (β WN) and network dissimilarity due to either species turnover (β ST) or rewiring (β OS) among and within habitat layer comparisons. Supporting Information S7. Species distribution across the habitat axis, partitioned by trophic levels/species groups. Colours indicate module affiliation, as in Figure 4. Supporting Information S8. Summary of versatility values (i.e. proportion of species switching modules, layers or habitats at least once) discriminated by species groups (italic values) within each trophic level (bold values). Supporting Information S9. Closeness centralities of species in forest (x axis) and grassland (y axis). Supporting Information S10. Summary of AICc classification for models with ΔAICc <2 (equally plausible). [file JANE-94-2205-s001.pdf]

Supporting Information for the paper "Across the edge: Spatial segregation drives community structure in tri-trophic multilayer networks at a forest–grassland edge"

***Supporting Information 1* (S1)**

Intralayer edges connected different species state nodes between trophic levels (vertical edges) and were weighted as the abundance of each prey species captured by each consumer species (consumer-prey interactions), or the number of cells of a given consumer-level species parasitised by a parasitoid species (host-parasitoid interactions). This was done to standardise interactions among parasitoids, as species may deliver from a single to hundreds of offspring per cell due to life-history traits. All intralayer edges connected the consumer level to the parasitoid or prey level, never linking a prey node directly to a parasitoid node. Interlayer edges also occurred between trophic levels but were based on intralayer edges, linking '2 species motifs' (interaction pairs), present in multiple layers (diagonal edges). We did so to (1) allow interlayer connections only between interaction pairs, ensuring better detection of state node module turnover (*i.e.* species interacting with different partners across layers will likely differ in their module associations) and (2) avoid single species modules, where several state nodes of the same species may form an individual module (commonly found in 'multiplex' multilayer structures, where interlayer edges connect state nodes from the same species). For example, given that a lower trophic level state node *X* has an intralayer edge to higher trophic level state node *Y* in layer *L1*, and the same interaction *XY* is present in the adjacent layer *L2,* state node *X*(*L1*) will have an interlayer edge to state node *Y*(*L2*), with an interlayer edge weight as follows:

$$\omega_{X(L1)\rightarrow Y(L2)}^{interlayer} = \frac{\omega_{XY}^{intralayer}(L2)}{|L1 - L2|} \qquad \text{(Eq. 1)}$$

where $\omega_{XY}^{intralayer}(L2)$ represents the weight of the intralayer link between nodes *X* and *Y* in layer *L2*. By doing so, the interlayer edges were weighted mirroring the spatial increase or decrease of the observed interaction degree at each layer (intralayer edges). This value was then divided by the number of layers separating the nodes (|*L1*-*L*2|, given that layers are named sequentially), to allow for further interlayer edges if interaction *XY* was not present in immediately adjacent layers, as follows:

$$\omega_{X(L_{origin})\rightarrow Y(L_{destiny})}^{interlayer} = \frac{\omega_{XY}^{intralayer}(L_{destiny})}{|L_{origin} - L_{destiny}|} \qquad \text{(Eq. 2)}$$

This allowed us to penalise edges between spatially distant layers while preserving interlayer edges between adjacent layers (which can be seen as positive, since the spatial amplitude of each layer is somewhat arbitrary). The calculation was applied to all subsequent interactions across layers (one layer pair at a time) and computed in both directions (Grassland → Forest and Forest → Grassland), enabling free interlayer movement along the habitat axis. Although the edges were undirected (able to flow vertically between trophic levels and diagonally between spatial layers), they were represented as bidirectional directed edges (one direction at a time, vertically and diagonally) to avoid restrictions imposed by the *Infomap* algorithm.

***Supporting Information 2* (S2)**

We computed multilayer closeness centrality for all physical nodes (species), by finding the shortest undirected path between the state nodes of any given species pair. However, the original mathematical definition of closeness does not apply to networks with isolated/disconnected nodes (Freeman, 1978), common in multilayer structures. Thus, we used the approach proposed by Opsahl (2010), for the closeness of a given species *i*:

$$closeness(i) = \frac{1}{(N-1)} \sum_{j \neq i} \frac{1}{d_{ij}} \quad \text{(Eq. 3)}$$

where d_{ij} is the shortest path between nodes *i* and *j*, and if they are disconnected from each other then $d_{ij} = \infty$. Mathematically, as $\frac{1}{\infty}$ tends to zero (in fact, zero *is* the output in the programming language *R*) it is possible to consider unreachable nodes and therefore penalise the metric for species in disconnected components. As d_{ij} can vary if state nodes *i* and *j* are within reach in multiple spatial layers, we modified the equation as:

$$closeness(i) = \frac{1}{(N-1)} \sum_{j \neq i} \frac{1}{d_{ij}^{min}} \quad \text{(Eq. 4)}$$

where $d_{ij}^{min} = min_{(L)}\left(d_{ij}^{(L)}\right)$, meaning the minimal shortest path between node *i* and *j* at any spatial layer (*L*) where both state nodes occur. Although shortest paths are usually computed for unweighted networks (*i.e.* the number of steps separating two nodes), we extended this measure for our weighted network by using average shortest distances proposed by Dijsktra (1959) to compute weighted shortest paths, and then counting the steps. Finally, as the final closeness value is sensitive to network size (especially when there are mesoscale topological structures such as habitats/spatial layers that may impact node isolation), we computed closeness within each habitat (forest/grassland, 3 layers each) and over the full multilayer network (6 layers), to standardize species effects at each of those structures. All shortest paths were calculated using the ‘igraph’ *R* package (Csardi & Nepusz, 2006).

Supporting Information 3 **(S3)**

We partitioned the centrality values of each cross-edge species by weighing their values within each habitat against their values over the entire network. To make matters easier, we will refer to closeness values within forest as “CF”, within grassland as “CG”, and over the full network as “CN”. As closeness values reflect the size of each network (species-wise), we converted CF/CG values to their CN counterpart by dividing their within-habitat closeness sum (Eq. 4, before the standardization) by 141 (the full set instead of the habitat set of species, minus the species itself). For single-habitat species, this new CF/CG value will be exactly the same as their corresponding CN value (as their contribution to CN centrality is only due to their effect within their habitat, and in the original CN calculation the same division by 141 was realized). For cross-edge species, however, the sum of the new CF/CG values will be marginally greater than its original CN value, as shortest paths to other cross-edge species are being measured twice (once for the CF structure, and once for the CG). To obtain the final partition of cross-edge species CN value, we:

(1) Divided their new CF/CG value by the CF/CG sum, rendering a regression coefficient for each habitat partition (the proportion of each habitat closeness);
(2) Multiplied their original CN value by both regression coefficients, to obtain the final habitat partition of closeness values (correcting for the CN values).

To address species' potential to bridge dynamics across habitats, we took advantage of the fact that the normalized closeness varies from 0 (no capacity to affect other species, all nodes are unreachable) to 1 (total capacity to affect other species, all nodes reachable within 1 step). Thus, by multiplying cross-edge species CF by their CG value, we effectively calculated their capacity to capture dynamics in the forest and transfer it to the grassland habitat, and *vice-versa* (species with high closeness in both habitats will have a greater potential, species with high closeness in only one habitat will have a smaller potential, and so on). Finally, as this “bridging potential” value is measured as a proportion of the species' total closeness (CF and CG sum), we multiplied the species CN value by this bridging potential to obtain the final bridging value scaled for the CN structure.

***Supporting Information 4* (S4)**

To address species' influence across the spatial/modular landscape, we followed Hackett et al. (2019) approach on Guimerà & Nunes Amaral (2005) work, allowing for the calculation of weighted connectivity metrics across the landscape (spatial layers) and interaction clusters (modules). This approach requires two indices, a within-degree one (z) and a participation coefficient (c). As species interact across multiple layers/modules with varying intensity, we can evaluate their influence within individual layers/modules (within-weight metrics) and across the spatial/modular landscape (cross-connectivity metrics):

- *Within-layer weight*: How influential the species is across all spatial layers where it occurs—that is, the proportion of all interactions in each layer involving the species, standardised across all layers in which it is present;
- *Within-module weight*: How influential the species is across all modules it was affiliated with—that is, the proportion of all interactions in each module involving the species, standardised across all modules in which it is present;
- *Cross-layer connectivity*: How evenly distributed are a species' interactions across the spatial layers it occurs;
- *Cross-module connectivity*: How evenly distributed are a species' interactions across the modules it was affiliated with;

All four metrics account for species observations in multiple modules or layers/habitats (through their state nodes), thus dividing the community into subsets of shared state nodes (for modules or layers). To measure how strongly a species *i* is connected in a focal module or spatial layer (structure *h*), we calculate its local within-weight z_{ih}:

$$z_{ih} = \frac{(k_{ih} - k_h)}{\sigma_{j \in V_h}(k_{jh})} \qquad \text{(Eq. 5)}$$

where k_{ih} represents the weight of species *i* in structure *h*, while k_h denotes the average weight of species occurring within structure *h*. The term $\sigma_{j \in V_h}(k_{jh})$ corresponds to the standard deviation of species weight in structure *h*, where V_h is the set of species present in the structure. By taking the weighted arithmetic mean of local within-weights z_{ih}, we define its final within-weight z_i:

$$z_i = \sum_h B_{ih} z_{ih} \qquad \text{(Eq. 6)}$$

where B_{ih} is the belonging coefficient of species *i* to structure *h*, representing the proportion of species *i*'s interactions that occur within structure *h*. The cross-connectivity c_i measures how evenly distributed the interactions of a given species are across the spatial/modular landscape:

$$c_i = 1 - \sum_h B_{ih}^2 \qquad \text{(Eq. 7)}$$

The value of c_i approaches 1 if interactions are evenly distributed across the landscape, whereas it tends towards 0 if interactions occur within a single layer/module.

***Supporting Information 5* (S5)**

We tested four different hypotheses 'families' to explain species influence across the network. For each of the following, we generated all possible combinations of fixed effects (predictor variables):

- *Spatial hypothesis.* Species' spatial dynamics explain most of their network-level influence (Fixed effects: within-layer and/or cross-layer).
- *Modular hypothesis.* Species' modular dynamics explain most of their network-level influence (Fixed effects: within-module and/or cross-module).
- *Global hypothesis.* A combination of spatial and modular dynamics explains most of a species' network-level influence (Fixed effects: within-layer and/or cross-layer, and within-module and/or cross-module).
- *Null hypothesis.* None of the predictors explains species network-level influence. The model includes only the regression intercept (i.e., the mean response), with no predictor variables.

We checked for correlations between all predictor pairs to avoid models with variance inflation factors (VIF) higher than 5. If such pairs were present, we extracted the principal component between those variables to be used as a predictor instead, thus avoiding multicollinearity.

For the random effects structure (species subgroups), we first considered models with only a random intercept varying within each random effect (trophic level and habitat). Next, we allowed each fixed effect, one at a time, to vary by a random effect (random intercept + random slope). This approach aimed to identify the single best predictor for each random effect grouping, resulting in a total of 41 models. Given the variation in fixed effects, models were fitted using the Maximum Likelihood (ML) method (Snijders & Bosker, 2011), and after model selection, refitted using Restricted Maximum Likelihood (REML) to avoid underestimation of model variance. We then: (i) built an Akaike Information Criterion table to select the models with smaller AICc; (ii) if the best models were from the same family (i.e. same fixed effects), we considered Burnham & Anderson (2002) findings on models with small AICc variation as equally plausible and thus averaged models with ΔAICc < 2 to better capture variation in fixed effects across similar models. Following this logic, we (iii) picked the model with the highest variation explained by fixed and random effects (Conditional 'R' Squared), to find the best random effects structure to represent our data. Finally, we calculated 95% confidence interval for each fixed effect predictor (considering the average model) to account for the overall relationships between species influence and weight/connectivity metrics. Confidence intervals including zero were not considered significant, as they highlight no consistent positive/negative relationship between the predictor and the response variable (Burnham & Anderson, 2022). We applied the same approach to each random effect predictor to examine how species' responses to predictors vary across different groupings (*Habitat*: single-habitat grassland/forest and cross-edge species; *Trophic level*: prey/consumer/parasitoid). Model building, selection and averaging were done using 'lme4' (Bates et al, 2023) and 'MuMIn' (Bartón, 2023) *R* packages (R Core Team, 2024).

Supporting Information 6 **(S6).** Summary of species composition dissimilarity (β_S), network dissimilarity (β_{WN}) and network dissimilarity due to either species turnover (β_{ST}) or rewiring (β_{OS}) among and within habitat layer comparisons. G is for grassland, F for forest in the *x-axis*, and numbers beside them are layers in order of distance from the edge.

Layer comparison	Spatial pair	Layer pair	β_S	β_{WN}	β_{ST}	β_{OS}
Within grassland	Interior - Edge	G3-G1	0.49	0.51	0.51	0
	Interior - Intermediate	G3-G2	0.50	0.53	0.50	0.03
	Intermediate- Edge	G2-G1	0.40	0.44	0.44	0
Within forest	Interior - Edge	F1-F3	0.45	0.54	0.39	0.14
	Interior - Intermediate	F2-F3	0.46	0.52	0.44	0.08
	Intermediate- Edge	F1-F2	0.27	0.32	0.23	0.09
Across edge	Edge - Edge	G1-F1	0.77	0.98	0.98	0.02
	Intermediate- Intermediate	G2-F2	0.78	1	1	0
	Interior - Interior	G3-F3	0.75	1	1	0

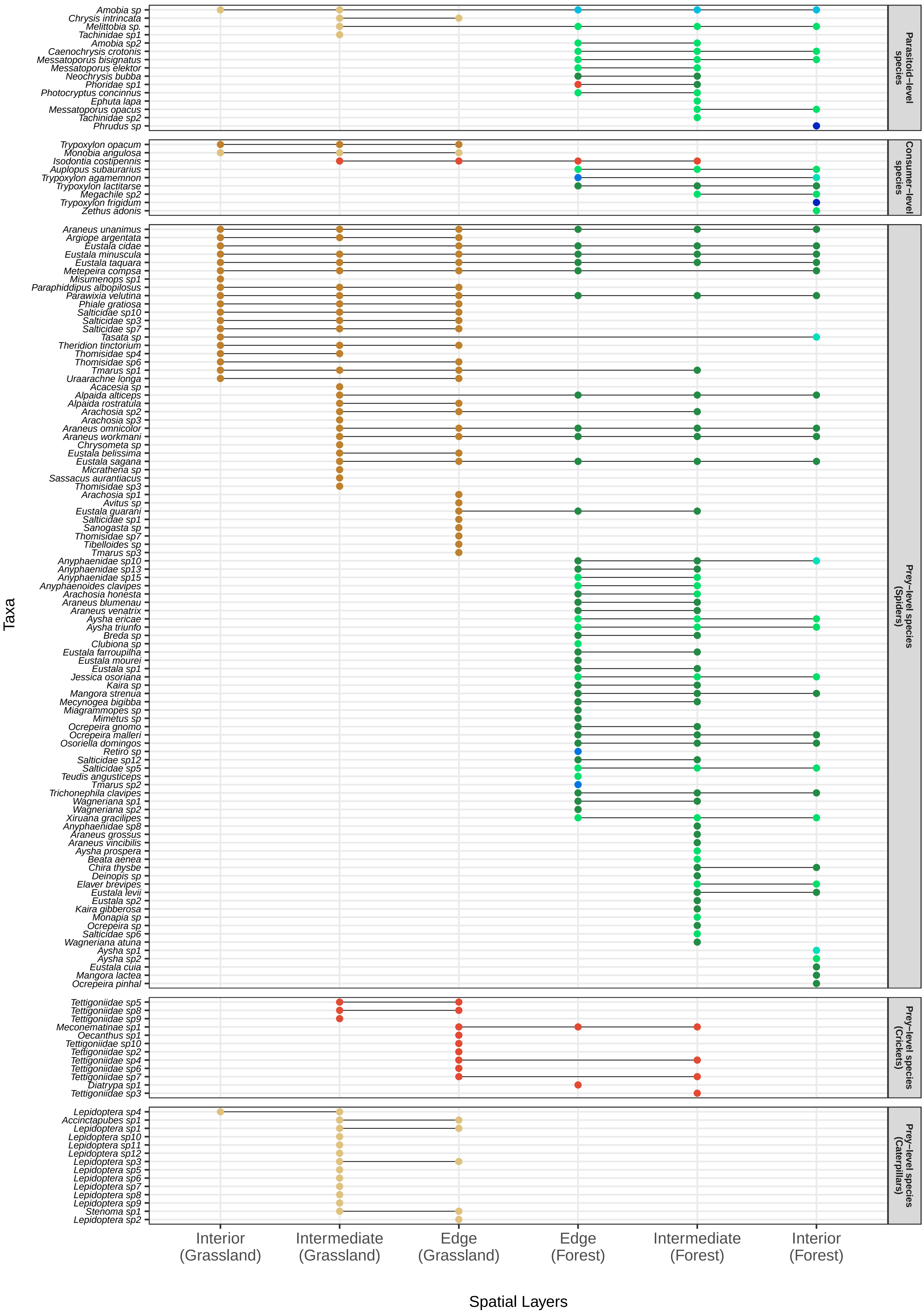

Amobia sp
Chrysis intrincata
Melittobia sp.
Tachinidae sp1
Amobia sp2
Caenochrysis crotonis
Messatoporus bisignatus
Messatoporus elektor
Neochrysis bubba
Phoridae sp1
Photocryptus concinnus
Ephuta lapa
Messatoporus opacus
Tachinidae sp2
Phrudus sp
Parasitoid–level species
Trypoxylon opacum
Monobia angulosa
Isodontia costipennis
Auplopus subaurarius
Trypoxylon agamemnon
Trypoxylon lactitarse
Megachile sp2
Trypoxylon frigidum
Zethus adonis
Consumer–level species
Araneus unanimus
Argiope argentata
Eustala cidae
Eustala minuscula
Eustala taquara
Metepeira compsa
Misumenops sp1
Paraphiddipus albopilosus
Parawixia velutina
Phiale gratiosa
Salticidae sp10
Salticidae sp3
Salticidae sp7
Tasata sp
Theridion tinctorium
Thomisidae sp4
Thomisidae sp6
Tmarus sp1
Uraarachne longa
Acacesia sp
Alpaida alticeps
Alpaida rostratula
Arachosia sp2
Arachosia sp3
Araneus omnicolor
Araneus workmani
Chrysometa sp
Eustala belissima
Eustala sagana
Micrathena sp
Sassacus aurantiacus
Thomisidae sp3
Arachosia sp1
Avitus sp
Eustala guarani
Salticidae sp1
Sanogasta sp
Thomisidae sp7
Tibelloides sp
Tmarus sp3
Anyphaenidae sp10
Anyphaenidae sp13
Anyphaenidae sp15
Anyphaenoides clavipes
Arachosia honesta
Araneus blumenau
Araneus venatrix
Aysha ericae
Aysha triunfo
Breda sp
Clubiona sp
Eustala farroupilha
Eustala mourei
Eustala sp1
Jessica osoriana
Kaira sp
Mangora strenua
Mecynogea bigibba
Miagrammopes sp
Mimetus sp
Ocrepeira gnomo
Ocrepeira malleri
Osoriella domingos
Retiro sp
Salticidae sp12
Salticidae sp5
Teudis angusticeps
Tmarus sp2
Trichonephila clavipes
Wagneriana sp1
Wagneriana sp2
Xiruana gracilipes
Anyphaenidae sp8
Araneus grossus
Araneus vincibilis
Aysha prospera
Beata aenea
Chira thysbe
Deinopis sp
Elaver brevipes
Eustala levii
Eustala sp2
Kaira gibberosa
Monapia sp
Ocrepeira sp
Salticidae sp6
Wagneriana atuna
Aysha sp1
Aysha sp2
Eustala cuia
Mangora lactea
Ocrepeira pinhal
Prey–level species (Spiders)
Tettigoniidae sp5
Tettigoniidae sp8
Tettigoniidae sp9
Meconematinae sp1
Oecanthus sp1
Tettigoniidae sp10
Tettigoniidae sp2
Tettigoniidae sp4
Tettigoniidae sp6
Tettigoniidae sp7
Diatrypa sp1
Tettigoniidae sp3
Prey–level species (Crickets)
Lepidoptera sp4
Accinctapubes sp1
Lepidoptera sp1
Lepidoptera sp10
Lepidoptera sp11
Lepidoptera sp12
Lepidoptera sp3
Lepidoptera sp5
Lepidoptera sp6
Lepidoptera sp7
Lepidoptera sp8
Lepidoptera sp9
Stenoma sp1
Lepidoptera sp2
Prey–level species (Caterpillars)
Taxa
Interior (Grassland)
Intermediate (Grassland)
Edge (Grassland)
Edge (Forest)
Intermediate (Forest)
Interior (Forest)
Spatial Layers

Supporting Information *7* **(S7)**. Species distribution across the habitat axis, partitioned by trophic levels/species groups. Colours indicate module affiliation, as in Figure 4.

***Supporting Information 8* (S8).** Summary of versatility values (i.e. proportion of species switching modules, layers or habitats at least once) discriminated by species groups (italic values) within each trophic level (bold values). Dashed lines set the boundaries for each trophic level.

Species group/level	Module versatility	Layer versatility	Habitat versatility	Nº of species
Caterpillars	*0*	*0.35*	*0*	*14*
Crickets	*0*	*0.41*	*0.25*	*12*
Spiders	*0.17*	*0.57*	*0.15*	*92*
**Prey-level**	**0.13**	**0.53**	**0.14**	**118**
Bees	*0*	*1.0*	*0*	*1*
Cricket-hunter wasps	*0*	*1.0*	*1.0*	*1*
Caterpillar-hunter wasps	*0*	*0.5*	*0*	*2*
Spider-hunter wasps	*0.2*	*0.8*	*0*	*5*
**Consumer-level**	**0.11**	**0.77**	**0.11**	**9**
**Parasitoid-level**	**0.2**	**0.73**	**0.13**	**15**

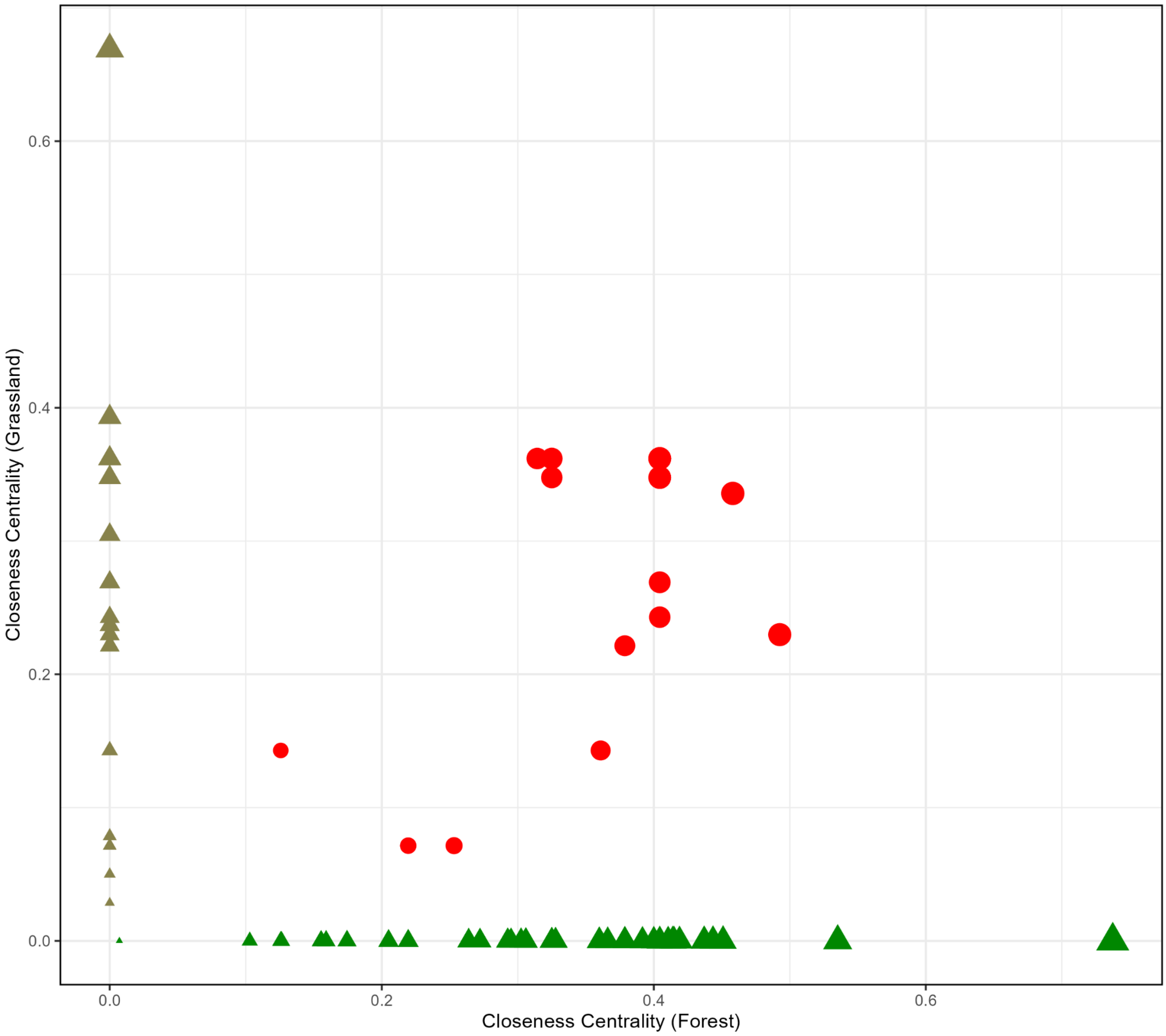

Supporting Information 9 **(S9)**. Closeness centralities of species in forest (x axis) and grassland (y axis). Point size reflects 'full network' closeness. Single-habitat species (triangles) align with axes (green or brown); cross-edge species (circles, red) have x|y > 0.

***Supporting Information 10* (S10).**

Two of our predictor variables (within-layer and within-module) were highly correlated (93%). To address this, we extracted their principal component to create a variable 'within-weight,' which represents a species overall dominance across all modules and layers in which they occur (*i.e.* the averaged proportion of all interactions involving the species in both of these structures). This change was only applied to our “global” models (thus avoiding multicollinearity between those predictors), whereas in the ‘modular’ and ‘spatial’ models the original predictors were considered instead.

We found 4 equally plausible models based on AICc criteria (ΔAICc < 2). All models were from the ‘global’ hypotheses, accounting for spatial and modular connectivity as predictors of species network influence. Given their similarity (in ΔAICc and fixed effects structure), we averaged those models to extract more robust parameter estimates across them (Figure 7a). For the same reason, we chose the model with the highest conditional R^2 to represent our random effects structure.

Summary of AICc classification for models with ΔAICc < 2 (equally plausible). *Global* hypotheses stand for all fixed effects (within-weight, cross-layer and cross-module). Bold cells indicate the chosen model, with the highest Conditional R^2.

Model structure	AICc	ΔAICc	Weight	Conditional R^2	Marginal R^2
Global hypothesis; Random intercepts; Random slope for cross-module across habitats; Random slope for within-weight across trophic levels;	-454.23	0	0.32	0.819*	0.294**
Global hypothesis; Random intercepts;	-453.83	0.4	0.26	0.812*	0.461**
***Global* hypothesis; Random intercepts; Random slope for cross-module across habitats; Random slope for cross-layer across trophic levels;**	**-453.09**	**1.14**	**0.18**	**0.837***	**0.371****
Global hypothesis; Random intercepts; Random slope for cross-module across habitats; Random slope for cross-module across trophic levels;	-452.91	1.32	0.16	0.814*	0.345**

*Represents the variation explained by all independent variables in the model (both fixed and random effects).
** Represents the variation explained only by the independent variables of fixed effects.

References (Supporting Information)

Bartón, K., 2023. MuMIn: Multi-model inference. R package version. Available at: https://cran.r-project.org/package=MuMIn.

Bates, D., Mächler, M., Bolker, B., & Walker, S., 2023. lme4: Linear mixed-effects models using 'Eigen' and S4. R package version.

Burnham, K.P., & Anderson, D.R., 2022. Model selection and multimodel inference: A practical information-theoretic approach. 2nd ed. Springer.

Csardi, G., & Nepusz, T., 2006. The igraph software package for complex network research. *InterJournal, Complex Systems*, 1695.

Dijkstra, E.W., 1959. A note on two problems in connexion with graphs. *Numerische Mathematik*, *1*(1), pp.269–271. Available at: https://doi.org/10.1145/3544585.3544600

Freeman, L.C., 1978. Centrality in social networks conceptual clarification. *Social Networks*, *1*(3), pp.215–239. ISBN 0-415-25108-7

Guimerà, R., & Nunes Amaral, L. A. (2005). Functional cartography of complex metabolic networks. *nature*, *433*(7028), 895-900. Available at: https://doi.org/10.1038/nature03288

Hackett, T. D., Sauve, A. M., Davies, N., Montoya, D., Tylianakis, J. M., & Memmott, J. (2019). Reshaping our understanding of species' roles in landscape‐scale networks. *Ecology Letters*, *22*(9), 1367-1377. Available at: https://doi.org/10.1111/ele.13292

Opsahl, T., Agneessens, F. & Skvoretz, J. Node centrality in weighted networks: Generalizing degree and shortest paths. Social networks 32, 245–251 (2010).

R Core Team, 2024. R: A language and environment for statistical computing. R Foundation for Statistical Computing.

Snijders, T.A.B., & Bosker, R.J., 2011. Multilevel analysis: An introduction to basic and advanced multilevel modeling. 2nd ed. Sage Publications.
